# Increased dosage of *AOX1* promoter-regulated expression cassettes leads to transcription attenuation of the methanol metabolism in *Pichia pastoris*

**DOI:** 10.1038/srep44302

**Published:** 2017-03-15

**Authors:** Elena Cámara, Nils Landes, Joan Albiol, Brigitte Gasser, Diethard Mattanovich, Pau Ferrer

**Affiliations:** 1 Department of Chemical, Biological, and Environmental Engineering, Escola d’Enginyeria, Universitat Autònoma de Barcelona, Bellaterra (Cerdanyola del Vallès) 08193, Catalonia, Spain; 2 Department of Biotechnology, BOKU - University of Natural Resources and Life Sciences Vienna, Muthgasse 18, 1190 Vienna, Austria; 3 Austrian Centre of Industrial Biotechnology, A-1190 Vienna, Austria

## Abstract

The methanol-regulated alcohol oxidase promoter (P_*AOX1*_) of *Pichia pastoris* is one of the strongest promoters for heterologous gene expression in this methylotrophic yeast. Although increasing gene dosage is one of the most common strategies to increase recombinant protein productivities, the increase of gene dosage of *Rhizopus oryzae* lipase (*ROL*) in *P. pastoris* has been previously shown to reduce cell growth, lipase production and substrate consumption in high-copy strains. To better assess that physiological response, transcriptomics analysis was performed of a subset of strains with 1 to 15 *ROL* copies. The macroscopic physiological parameters confirm that growth yield and carbon uptake rate are gene dosage dependent, and were supported by the transcriptomic data, showing the impact of increased dosage of *AOX1* promoter-regulated expression cassettes on *P. pastoris* physiology under steady methanolic growth conditions. Remarkably, increased number of cassettes led to transcription attenuation of the methanol metabolism and peroxisome biogenesis in *P. pastoris*, concomitant with reduced secretion levels of the heterologous product. Moreover, our data also point to a block in *ROL* mRNA translation in the higher *ROL*-copies constructs, while the low productivities of multi-copy strains under steady growth conditions do not appear to be directly related to UPR and ERAD induction.

The methylotrophic yeast *Pichia pastoris (Komagataella sp.*) is a well-established host system for protein production, with an increasing relevance for the production of commercial recombinant products^1^. Among several factors, the presence of the strong and tightly regulated *AOX1* promoter (P_*AOX1*_) has contributed to the popularity of this expression system. In the presence of methanol, the P_*AOX1*_ is strongly induced leading to a high amount of the enzyme alcohol oxidase^2^,^3^,^4^. Consequently, P_*AOX1*_ is the most commonly used promoter for recombinant protein production in *P. pastoris*^5^,^6^. To further increase recombinant protein production in P_*AOX1*_-based systems, several strategies have been attempted, such as optimisation of cultivation conditions and feed strategies^7^,^8^, co-expression of folding and secretion factors^9^,^10^,^11^,^12^ or optimising the properties of the foreign nucleotide sequence (codon usage)^13^,^14^,^15^. In this context, the insertion of multiple recombinant gene copies has been successfully used^16^,^17^,^18^,^19^. However, these studies also reported that increased recombinant gene dosage beyond a certain threshold often resulted in lower productivities and a severe negative impact on cell growth, reduced methanol consumption and decreased cell viability, i.e. reflecting a metabolic burden effect. To date, most of the studies aiming to understand the physiological responses and adaptations related with the high-level production of recombinant proteins in multi-copy strains focused on the stress responses elicited by the high product formation. For secreted proteins, in particular stresses triggered by the accumulation of recombinant proteins in the secretory pathway, that is, the Unfolded Protein Response (UPR), the endoplasmic reticulum (ER) oxidative stress and the ER-associated protein degradation (ERAD) pathway were analysed^20^,^21^,^22^. Also, there is increasing evidence that increased recombinant gene dosage leads to a metabolic burden effect at the level of central carbon metabolism, thus pointing at a limitation in the carbon and energy supply^23^,^24^. Although systems-level approaches to understand the physiology of recombinant *P. pastoris* are increasingly being used^4^,^25^,^26^,^27^,^28^,^29^,^30^, genome scale analysis to understand the impact of increased recombinant gene dosages on the physiology of *P. pastoris* has largely been missing, particularly for P_*AOX1*_-based systems. In a previous study^16^, a series of strains harbouring different copy numbers of a *Rhizopus oryzae* lipase gene (*ROL*) under control of the P_*AOX1*_ was constructed and characterised, showing decreased product and biomass yields, as well as altered substrate specific consumption rates in the multi-copy strains in relation to the single copy strain.

In the present study we performed global transcriptomic analyses of a set of multi-copy strains to gain better insight into the impact of heterologous gene (*ROL*) dosage on the metabolic network of *P. pastoris* under methylotrophic conditions. To minimize effects of other variables (e.g. specific growth rate) these analyses were carried out in continuous cultures under steady state conditions using a mixture of glycerol and methanol as carbon source to allow induction of *ROL* expression. Furthermore, Rol production was monitored both at transcriptional (Digital Droplet PCR) and translational (intra/extracellular enzyme activity and Western Blot analysis) level.

## Materials and Methods

### Strain construction

A series of *P. pastoris* X-33 (Invitrogen, Carlsbad, CA, US) derived strains, with an increasing number of copies of a *Rhizopus oryzae* lipase gene (*ROL*) cloned into the pPICZαA (Invitrogen, Carlsbad, CA, US) vector were used in this study. Methodology of strain construction and characterisation was reported in a previous study^16^. Briefly, different pPICZαA-based vectors carrying one, two or four copies of *ROL* were obtained by means of the construction of *in vitro* multimers, and subsequently introduced into *P. pastoris* X-33 by electroporation to generate the multi-copy strains. A representative clone assortment of different copy range carrying 2, 4, 8 and 15 copies (named 2C, 4C, 8C, and 15C, respectively) was selected to further transcriptional characterisation. The untransformed X-33 strain (0C) was used as a negative control, whereas a strain containing one copy of *ROL* previously obtained by^31^ was selected as a single copy strain (1C).

### Chemostat cultures

Triplicate chemostat cultures were carried out at a working volume of 0.35 L in a 1-L vessel bioreactor (DASGIP^®^ Parallel Bioreactor Systems, Eppendorf, Hamburg, Germany) for each strain. As seed culture, a 0.5-L shake flask containing 50 mL of YPD-Zeo medium (per litre: 10 g yeast extract, 20 g peptone, 20 g glucose, 100 mg Zeocin) was inoculated with a cryostock of the *P. pastoris* strain to an optical density at 600 nm (OD_600_) of 0.3 and incubated for approximately 24 h at 25 °C and 180 rpm at an Infors Orbitron shaker (Infors AG, Bottmingen, Switzerland) to inoculate a volume of 0.4 L of batch media. At the end of batch phase (reaching a final OD_600_ of 50) the chemostat culture was started at a dilution rate (D) of 0.1 h^−1^, by feeding a defined growth medium (controlled by weighing scales) containing 25 g L^−1^ of glycerol/methanol mixture (60% glycerol/40% methanol, w/w) as a carbon source. This choice was based on the interest and potential of mixed substrate feeds for improvement of high cell density fed-batch cultivation processes for heterologous protein production purposes^32^,^33^ as well as to facilitate the comparison of datasets of this study with those previously reported mixed-carbon chemostat studies using the same reference Rol-producing strains^16^,^32^,^34^. Culture media for the batch and continuous culture phases were the same as previously described^16^. Antifoam (Glanapon 2000 Konc, Bussetti, Vienna, Austria) was added constantly at a rate of 13 μL h^−1^. Cultivation temperature was kept constant at 25 °C, pH was controlled at 5 with 10% ammonia and the dissolved oxygen concentration was kept at 20% by controlling the stirrer speed until a maximum of 1250 rpm at a constant airflow of 21 L h^–1^. Samples were taken after at least five residence times when steady state conditions were reached.

### Analytical procedures

#### Biomass determination

Chemostat cell biomass was monitored by measuring the OD_600_. Biomass concentration was also determined as dry cell weight (DCW) in triplicate for each sample. A volume of 5 mL of culture broth was centrifuged at 4100 rpm for 5 min, and then resuspended with 5 mL of deionized water (DI water). Cells were washed twice and dried in pre-weighted beakers at 105 °C during 24 h.

#### Lipase activity determination

The lipolytic activity assay was carried out in triplicates for each independent experiment as previously described^35^.

#### Cell disruption

Intracellular lipase activity was measured after disrupting cells mechanically using the method described by García-Ortega and co-coworkers^36^. A soluble and an insoluble cell extract were obtained, corresponding to cleared cell lysate and membrane-associated fraction, respectively. Cell disruption efficiency was calculated as follows (Equation 1):


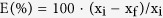


where E (%) is the degree of disruption; x_i_, the number of cells before disruption; x_f_, number of non-disrupted cells after disruption. Measures were performed from samples of three independent experiments for each strain.

#### Total protein determination

Quantification of total protein content of supernatant and lysate fractions was determined with the Pierce BCA Protein Assay Kit, according to the manufacturer’s instructions. Bovine serum albumin (BSA) was used as the protein standard for the calibration curve. Absorbance was measured in 96-well plates using the spectrophotometer Multiskan™ FC (ThermoFisher Scientific, Waltham, MA, US). All reagents were purchased from ThermoFisher Scientific (Waltham, MA, US). Determinations were performed in duplicates for each independent experiment.

#### Metabolite quantification

Glycerol, methanol and other potential extracellular compounds in the chemostat samples were analysed as previously described^16^. Duplicate analyses were performed for each independent experiment.

#### Fatty acid composition

Quantification of the total content of fatty acids in the chemostat samples was carried out by BIOCRATES Life Science AG (Innsbruck, Austria). Briefly, lyophilized cell samples from each chemostat were treated with methanolic HCl solution at high temperature for a prolonged time period to completely convert both bound and free fatty acids into their methyl esters representing the total fatty acid (TFA) content. The content of individual fatty acids in samples were determined as their corresponding methyl ester derivatives (FAME’s) using gas chromatography coupled with mass spectrometric detection (Agilent 7890 GC/5975 MSD) after derivatization. External standard calibration curves were used to calculate the corresponding concentrations.

### Rol quantification by Western Blot

Cell culture supernatant, soluble and insoluble lysate fractions were subjected to Western Blot (WB) analysis to determine *R. oryzae* lipase (Rol) content. To obtain reliable quantitative data, guidelines recommended by Taylor and co-workers^37^ were followed. Samples were diluted 3:1 in Laemmli buffer and heated at 95 °C during 10 min, followed by 10 min on ice. Samples were run in a sodium dodecyl sulphate–polyacrylamide gel electrophoresis (SDS–PAGE), 12%, carried out in a Mini-PROTEAN II apparatus using Mini-PROTEAN^®^ TGX Stain-Free™ Protein Gels, following the standard procedures recommended by the manufacturer. Precision Plus Protein™ Unstained and All Blue Standards were used as molecular weight markers, for SDS-PAGE and WB membrane visualization, respectively. Gel visualization was performed after UV activation using the Gel Doc EZ System. To perform Western blots after SDS-PAGE, proteins were transferred to a nitrocellulose membrane (10 min, 2.5 A, 25 V) using the Trans-Blot^®^ Turbo™ Transfer System. The membrane was blocked during 1 h incubation in 5% skim milk, 1 mL Tween 20, and then incubated with a 1:10000 mouse anti-Rol antiserum (obtained from the Servei de Cultius Cel·lulars, Producció d’Anticossos i Citometria, Universitat Autònoma de Barcelona, Bellaterra, Spain) for 1 h. After washing, the membrane was incubated with 1:1000 HRP-conjugated polyclonal anti-mouse IgG (Sigma Aldrich, St Louis, MO, US) as a secondary antibody during 1 h. Detection was carried out by incubating the membrane for five minutes with Clarity™ Western ECL Substrate. Further image analysis and quantification was performed with Molecular Imager^®^ChemiDoc™XRS System and the software ImageLab v.5.2. Quantifications were carried out from samples of three independent experiments for each strain. If not otherwise stated, all reagents were obtained from Bio-Rad (Hercules, CA, US).

### ROL transcriptional levels determination by Digital Droplet PCR

*ROL* expression levels were quantified in the extracted total RNA (see section on microarrays below) using Digital Droplet PCR. Therefore, cDNA was synthesized using the iScript™ cDNA Synthesis kit (Bio-Rad, Hercules, CA, US) following the manufacturer’s instructions, and Digital Droplet PCR (ddPCR) was subsequently performed. To normalise the data, the house-keeping gene β-actin (*ACT1*) was selected. For cDNA amplification, a set of *ROL* and *ACT1* primers were designed (Supplementary Table S1). Each PCR reaction mixture was prepared in 20 μL final volume, containing 10 μL of QX200™ ddPCR™ EvaGreen Supermix, 200 nM of forward primer, 100 nM of reverse primer, 0.4 ng of cDNA, and the required amount of Dnase/Rnase-free water. Droplet formation was carried out using the Droplet Generator QX200™ and further transferred into a 96-well plate. Reactions were incubated at 95 °C for 10 min and then subjected to a first step of denaturation (94 °C, 30 s) followed by an annealing/extension step (60.2 °C, 1 min, for *ROL* primers; 56.5 °C, 1 min, for *ACT1* primers) for a total of 40 cycles. Droplet detection was completed using the QX100™ Droplet Digital Reader and the software Quantasoft v. 1.7.4.0917. *ROL* expression was calculated for each sample in duplicate, normalising the data by calculating the ratio between positive droplets of *ROL* and *ACT1*. Reagents for ddPCR were purchased to Bio-Rad (Hercules, CA, US), whereas primers were synthesized by Sigma Aldrich (St Louis, MO, US).

### ROL copy number determination by Digital Droplet PCR

To verify the *ROL* gene dosage stability, biomass samples of the chemostat culture were taken after five residence times, once the steady state was achieved. Gene dosage was analysed by ddPCR using the method previously described^16^.

### Statistical analysis

Three chemostat cultures (biological replicates) were performed for each strain from independent pre-cultures. Growth physiological data was verified using standard data consistency and reconciliation procedures as previously described^16^. Data are expressed as mean ± standard deviation (SD). Statistical analyses of analytical datasets were carried out using ANOVA followed by the unpaired Student’s *t*-test using Microsoft’s Excel software. A p-value lower than 0.05 was considered statistically significant.

### RNA extraction and microarray hybridization

For transcriptomics analyses, total RNA of the three independent biological replicates per strain cultivated in chemostat was used. Chemostat samples were immediately added in a 2:1 ratio to a precooled fixing solution (5% v/v phenol in ethanol absolute) and centrifuged at 10,000 × g for 1 min. Pellets were stored at −80 °C before further processing. RNA isolation was performed using TRI reagent according to the suppliers’ instructions (Ambion, CA, US). RNA integrity and concentration were analysed using RNA Nano chips (Agilent, Santa Clara, CA, US) and a Nanodrop (Thermo Scientific™, Waltham, MA, US), respectively. Labelling, hybridization to the in-house designed *P. pastoris* specific oligonucleotide arrays (AMAD-ID 034821, 8 × 15 K custom arrays, Agilent) and scanning were done according to Agilent’s Two-Colour Microarray-Based Gene Expression Analysis protocol (Low Input Quick Amp Labelling Kit). All samples were labelled in a dye-swap manner and hybridized against a reference cDNA, which was generated from a pool of cells grown at various culture conditions (described in ref. 26). Normalization steps and statistical analysis of microarray data included removal of colour bias using locally weighted MA-scatterplot smoothing (LOESS) followed by an array normalization using the “Aquantile” method. The microarray data was not background corrected. The resulting p-values were adjusted for multiple testing using the method of Benjamini and Yekutieli^38^. For each strain, log_2_ fold change (FC) values were calculated against the non-expressing control strain (0C), using the limma package of the R-project^39^. For identifying differentially expressed genes, a fold change cut-off of at least −0.58 > Log_2_FC > 0.58 (equivalent to a fold change of 1.5) and an adjusted *P*-value < 0.05 were applied (Supplementary File S2). Microarray data are available in the ArrayExpress database (http://www.ebi.ac.uk/arrayexpress) under accession number E-MTAB-4582.

### Microarray data analysis

#### Venn diagrams

Venn diagrams were created using the web-based tool http://bioinformatics.psb.ugent.be/webtools/Venn/.

#### Clustering analysis

Clustering analysis of microarray data was performed in order to group regulated genes with similar expression patterns. Analysis was performed using log_2_FC values with the k-means algorithm^40^ and Euclidean distance implemented in Genesis software v.1.7.7^41^.

#### Principal Component Analysis (PCA)

Principal Component Analysis (PCA) was used to identify hidden pattern in the data set and identify the correlation among the variables^42^. Analysis was carried out using the with the Excel plug-in XLSTAT.

#### Functional enrichment analysis

Networks of GO/KEGG enriched terms versus control strain were visualized using the Cytoscape (v.3.2.1) plug-in ClueGO (v. 2.1.7)^43^. The ontology and annotation files for GO enrichment analysis were downloaded at 14.06.2016 whereas KEGG pathway database was released on 20.06.2016. The GO tree interval ranged from 8 to 12, and the minimum number of genes per cluster was set to two. Two-sided hypergeometric test was used to identify overrepresented GO and KEGG pathway terms with a significance level at 0.05 and Benjamini-Hochberg method (with a kappa score of 0.4) was used for the correction of false discovery rate. Doughnut representations of main functional categories in each strain were performed with Microsoft’s Excel software. GO tree level was limited from 7 to 10, with a minimum number of three genes per cluster and a minimum 5% of representation from the total associated genes with the GO term.

## Results and Discussion

### Multi-copy ROL strains show reduced cell growth and methanol consumption

A set of 6 clones of *P. pastoris* carrying a different copy number of *ROL*, previously selected from a collection of transformants^16^ was grown in glycerol-methanol chemostat cultures at a dilution rate of 0.1 h^−1^. Under these conditions, cultivations were glycerol-limited, thereby allowing for derepression and methanol induction of the methanol metabolism genes. By comparing different growth parameters (Table 1), a clear physiological impact could be observed as the *ROL* dosage increases, consistent with the previously reported results^16^. Specifically, biomass concentrations were reduced about 30–35% in the multi-copy strains compared to the non-expressing control 0C and the one copy clone 1C, also reflected in a significant (5–15%) decrease in biomass yields. In terms of substrate consumption, the glycerol consumption rate was 1.5-fold higher for these strains compared to the 0C control strain, whereas the specific consumption of methanol dropped sharply from −2.01 mmol g^−1^ h^−1^ (reference strain 0C) to −1.08 mmol g^−1^ h^−1^ for the 15-copy strain (and rates even lower for the 4C and 8C strains), accompanied by significant residual amounts of methanol in the culture media (Table 1 and Supplementary Table S3), reaching up to about 7.5 g/L in the 8C strain. Nonetheless, a growth-inhibitory effect of residual methanol on cell growth was discarded, as chemostats of multi-copy strains were stable (in terms of biomass, Rol and extracellular metabolites concentrations) after more than five bioreactor residence times. Also, ROS levels were insignificant and no extracellular formaldehyde accumulation was detected under steady growth conditions of multi-copy strains, further supporting that residual methanol concentration were below toxicity levels under such conditions^16^. Moreover, such reduced biomass yield of Rol multi-copy strains had also been previously observed in our previous small scale (shake flask) studies where this set of strains (included the 0C and 1C reference strains) were typically grown under methanol excess-conditions^16^.

### Lower ROL levels in high copy strains may be due to inefficient ROL transcription

**Figure 1:**
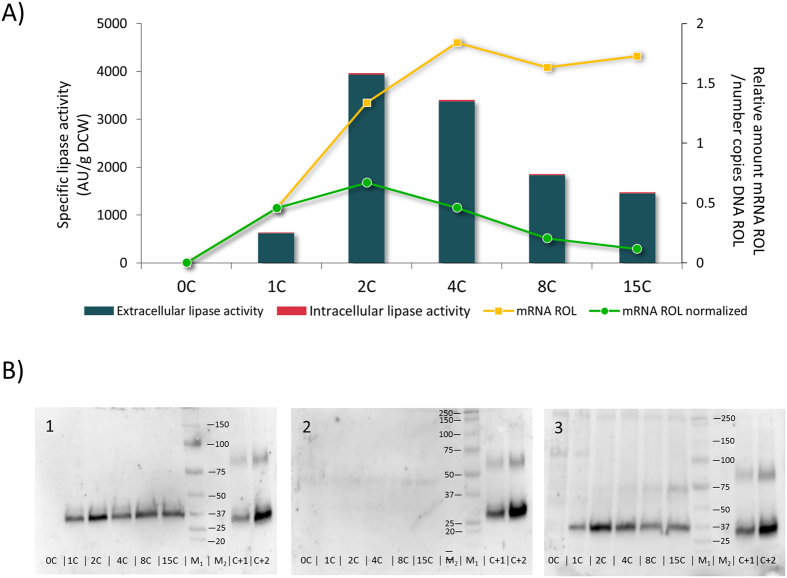
Analysis of Rol production at different stages. (**A**) In bars, extracellular and intracellular lipase activity (defined as units of lipase activity per g of Dry Cell Weight) of recombinant strains. Line graphs indicate mRNA expression levels. (**B**) Representative Western Blot analysis of different cellular fractions at the end of chemostat cultures. In membrane 1 samples 2C and 4C were diluted 1:2 to avoid signal saturation. Calculated values are provided in Supplementary File S4. 1. Samples of cell culture supernatant. 2. Samples of the soluble fraction of lysates. 3. Samples of the insoluble fraction of lysates. M_1_: visible marker; M_2_: unstained marker. C+1, C+2: positive controls, 9 ng and 18 ng of purified Rol, respectively.

Quantification of lipase production in *P. pastoris* strains was performed at different levels. Firstly, lipase secretion was measured as lipolytic activity in the culture supernatant. The specific extracellular lipase activity (UA/g DCW) profile obtained for the set of strains (Fig. 1A) was in agreement with the previously reported results^16^. Briefly, an optimum production level was achieved in cultivations of the 2C strain, whereas any further increase in *ROL* gene dosage led to lower specific lipase activities. In particular, the specific lipase activity levels achieved by the 2C strain were 6-fold higher compared to the single copy strain, while strains carrying higher *ROL* dosages (8C and 15C) exhibited lower increases comparing to the 1C (3-fold and 2-fold, respectively). Notably, intracellular lipase activity (measured in the soluble fraction of cell extracts) only represented about the 2% of the total activity produced by the strains, irrespective of their *ROL* gene dosage. Secondly, in order to determine whether intracellular and extracellular lipase activity profiles were correlated with Rol content (i.e. Rol mass), soluble and insoluble fractions of cell lysates, together with culture supernatants, were analysed by Western Blot. No lipase accumulation was detected in the soluble fraction of cell lysates, as observed in Fig. 1B. Conversely, significant amounts of lipase were detected in the insoluble fraction of cell lysates (i.e. membrane-associated fraction which contains secretory pathway organelles) (Supplementary Table S4). Nonetheless, there is no apparent difference between high and low copy number strains. In all tested strains, the lipase content in the insoluble cell fraction represented 30–40% of the total Rol mass (i.e. intracellular + extracellular content) detected by Western Blot analysis. Although extracellular protease activity was not measured in supernatant fractions, operational conditions (pH, temperature, nitrogen source) were selected to minimize cell lysis and protease secretion as previously described^44^. Furthermore, no degradation bands were detected in Western Blot analyses of intracellular fractions (Fig. 1B), reinforcing the hypothesis that proteolytic degradation (e.g. through the ERAD pathway) is not the major bottleneck in the higher *ROL* copy strains. Also, the constant relation between extracellular Rol content and extracellular lipolytic activity in all the strains indicates that there is no lipase inactivation. Consistently, Rol specific activity levels (i.e. UA Rol/mg total secreted protein) in the supernatant were correlated with the relative Rol amounts (mg secreted Rol/mg total secreted protein), being 0.5%, 7% and 3% for the single copy, the 2-copy strain and the higher copy strains, respectively. Overall, these observations indicate that intracellular Rol retention is not the main cause of decreased extracellular Rol levels at higher *ROL* gene dosages (4, 8 and 15-copy strains). This is in contrast to previously reported observations, where higher copy strains accumulated larger amounts of recombinant protein inside the cell than lower copy strains^20^,^45^. Similarly, previous studies with *P. pastoris* expressing *ROL* under the *FLD1* promoter showed that *ROL* overexpression in fed-batch cultures results in a significant Rol accumulation (up to 700 UA Rol/mg of total cell protein) in the soluble cell fraction of cell lysates after the first 55 h of induction^46^ (i.e. three orders of magnitude higher than in the present study), resulting in the transient induction of UPR at the initial stages of the fed-batch (inducing) phase^47^,^48^. Nonetheless, the expression system, bioreactor mode of operation and inducing conditions between these studies are not comparable to each other.

Regarding *ROL* transcriptional levels, a 4-fold increase between the 1C and the 4C strains can be observed (Fig. 1A), whereas a further increase of the *ROL* gene dosage in strains 8C and 15C does not result in higher mRNA levels. These results are in agreement with several previously reported cases where such a plateau-like correlation was observed^22^,^45^,^49^,^50^. Nevertheless, this pattern does not seem to be a general rule for P_*AOX1*_-based systems, as other studies pointed at a positive correlation between gene copy number and mRNA level when increasing gene dosage^20^,^51^. Strikingly, normalization of mRNA levels with the number of DNA copies of *ROL* (i.e. transcription efficiency expressed as amount of *ROL* mRNA transcribed per copy of *ROL* gene) of each strain showed an inverse relation between transcriptional efficiency and *ROL* gene dosage beyond 4 copies of this gene (Fig. 1A). This points to a transcriptional limitation of *ROL* expression as its copy number is increased, rather than a major limitation in folding and/or secretion. Importantly, the *ROL* copy number was verified routinely at the end of all chemostat cultures (after 5 residence times, equivalent to 7 cell generations), and no loss of *ROL* gene copies was detected in any of the strains (data not shown), thereby discarding the possibility that lower expression levels observed in higher *ROL* copy strains were the result of genetic instability (loss of expression cassettes) along the generations in chemostat cultivations. Notably, while *ROL* mRNA reached a plateau beyond four copies of *ROL*, enzyme levels decreased as copy number was increased from 4 to 15C, therefore also pointing at a limitation in translation, as discussed below.

### Global transcriptional profiling of ROL multi-copy strains

**Figure 2:**
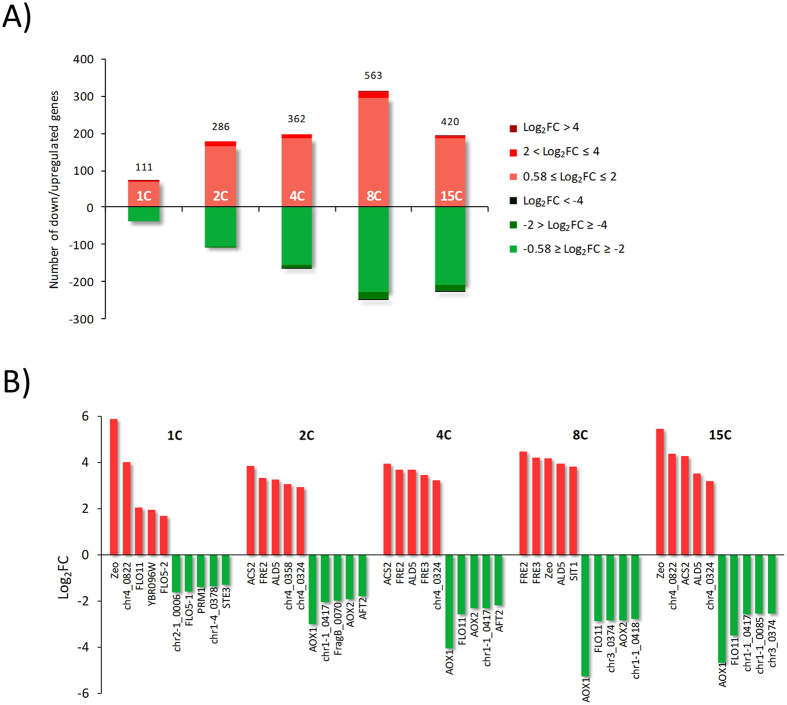
Global transcriptomic analysis of regulated genes in *ROL* strains. Regulated genes comprise those genes with a log_2_FC threshold of 0.58 comparing with the control strain and an adjusted p-val ≤ 0.05. (**A**) Number of regulated genes in each strain. Negative values indicate downregulated genes (green colour), whereas positive values designate upregulated genes (red colour). The number above the bars denotes the number of total regulated genes in each strain. (**B**) Classification of the most regulated genes.

Expression levels of 707 different genes significantly changed (Log_2_FC ± 0.58, adjusted p-value < 0.05) in at least one of the Rol producing strains when these were compared to the non-expressing control strain (Supplementary File S2). The total number of regulated genes generally increased with the *ROL* gene dosage except for the 15C strain, which showed a lower number of regulated genes in relation to the 8C strain (Fig. 2A). Additionally, the fraction of downregulated genes increased in the high copy strains, from 34% for the 1C to 54% of downregulated genes in 15C. Four out of the most regulated genes in the strains 2C and 4C (best producing strains) were identical (Fig. 2B). *ACS2*, coding for an acetyl-coA synthetase that acts as a nuclear source of acetyl-coA for histone acetylation, was the most upregulated gene in these strains, with a higher log_2_FC in the multicopy strains. *FRE2* and *FRE3*, encoding ferric and cupric reductases that facilitate the metal uptake in the cell, were also upregulated in all the strains, along with the aldehyde dehydrogenase gene *ALD6-2*. Unexpectedly, in all the multi-copy strains, the alcohol oxidase gene *AOX1* was the most downregulated gene. A negative regulation in the multi-copy strains was also observed for the acetate transporters encoded by PAS_chr1-1_0417 and PAS_chr1-1_0418. Strikingly, the gene annotated as flocculin gene *FLO11* (a GPI-anchored cell surface glycoprotein required for pseudohyphae formation and flocculation in *Saccharomyces cerevisiae*) was highly upregulated in the strain 1C, while it was downregulated in the multi-copy strains, particularly in the 4C, 8C and 15C strains. However, no apparent morphological changes were observed under the microscope among the strains (data not shown).

To evaluate the impact of *ROL* dosage on the gene expression pattern, the single copy strain (1C), the best producing strain (2C) and the strain with the highest *ROL* gene dosage (15C) were analysed in Venn diagrams. Data in Supplementary File S5 shows that a small set of genes was regulated in all the strains. Regarding the upregulated genes, 14% of 265 genes were common for all the three strains, comprising basically the fatty acid synthesis and β-oxidation. On the other hand, 7% of 247 genes were downregulated in all the strains, related with acylglycerol metabolism, pheromones and cell signalling (Supplementary File S6). While around 30% of regulated genes were shared between 2C and 15C strains (Supplementary File S6), the most prominent group was formed by genes only regulated (either up or downregulated) in the 15C strain. To further evaluate the variation among all the strains, Principal Component Analysis (PCA) was performed (Supplementary File S5). First and second principal components (PC1 and PC2) explained together 93% of the variability, grouping all the multi-copy strains separated from the 1C strain. Representation of the PC2 and PC3 (Supplementary File S5) allowed to arrange the multi-copy strains, showing the different behaviour of the 8C strain, with the highest number of regulated genes.

### Functional enrichment analysis

**Figure 3:**
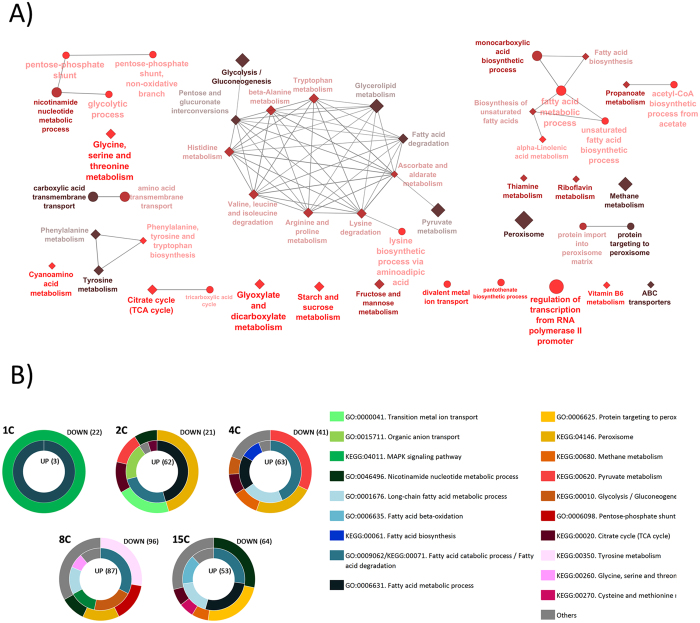
Functional analysis of regulated genes. (**A**) Network representation of enriched functional categories amongst *ROL* expressing strains, generated by ClueGO. Node size reflects the number of genes associated to a term whereas the gradient colour indicates the significance level (Bonferroni-Hochberg correction). GO and KEGG databases were used, represented as circles and diamonds, respectively. Individual networks are grouped by metabolic pathways. (**B**) Doughnut representations of main functional categories by strain. Outer and inner circles refer to down- and upregulated genes, respectively. Number in brackets indicates total amount of annotated genes in each condition.

Functional enrichment analysis of the 707 regulated genes was performed to identify the main biological processes involved and to recognize the relationships among them. ClueGO software was used with Gene Ontology (GO) and Kyoto Encyclopaedia of Genes and Genomes (KEGG) databases (Fig. 3A and Supplementary File S7). Statistically overrepresented processes (p-value < 0.05, with Benjamini-Hochberg correction) were grouped in 6 categories: 1) metal/polyamine transport (39 genes), 2) peroxisome/methanol metabolism (56 genes), 3) amino acid metabolism (74 genes), 4) fatty acid metabolism (50 genes), 5) regulation of transcription (39 genes), and 6) central carbon metabolism processes/redox reactions (82 genes). In addition, we also analysed the expression patterns of UPR-regulated genes among the series of strains. An overview of the regulated processes in each strain is represented in Fig. 3B. As it can be observed, the fatty acid catabolic process was enriched among the up-regulated genes in all the producing strains. However, only the multi-copy strains (2C, 4C, 8C and 15C) showed a wide variety of processes involving fatty acids being regulated. The main category of genes being downregulated in all the multi-copy strains was related to peroxisomes. In addition, a negative regulation of the methanol pathway was overrepresented in the best producing strains (2C and 4C). Furthermore, only in these strains a downregulation of tricarboxylic acid cycle (TCA) and pyruvate metabolism genes was also detected. Finally, the nicotinamide metabolic process was found to be regulated in the multi-copy strains, particularly in the case of the 15C strain.

**Figure 4:**
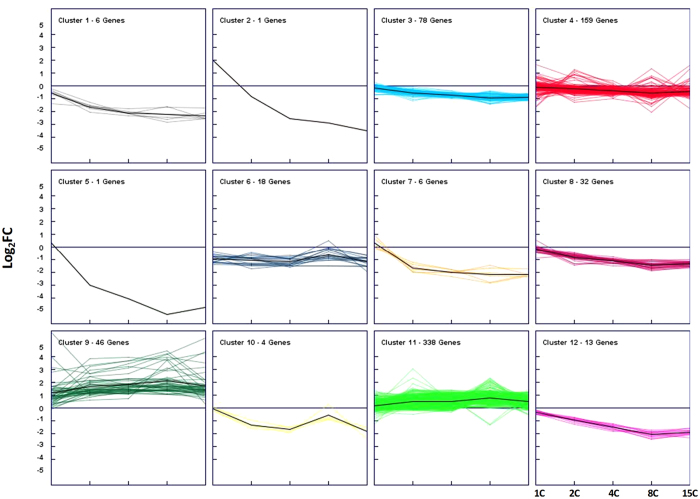
Cluster analysis of regulated genes in expressing strains. Expression profiles were grouped in 12 categories using the k-means clustering algorithm to divide the population in the main expression trends. Mean expression of each plot is represented by a black line.

Regulated genes were clustered using a k-means analysis to classify them in 12 different groups according to their expression patterns (Fig. 4). Seven clusters displayed an evident negative correlation as the *ROL* copy number increased in the strains (clusters 1, 2, 3, 5, 7, 8 and 12). Among these groups, clusters 1, 2 and 3 comprised the most regulated genes previously shown in Fig. 2A (lower panel). Cluster 3 was significantly enriched in terms of genes related to peroxisomes, pyridine nucleotide process, thiamine metabolism, metal iron transport and glycolysis (Supplementary File S8). Cluster 8 and cluster 12, also with a strong negative regulation profile, contained, among others, genes related with peroxisome, methanol metabolism and the glyoxylate cycle. Concerning the clusters with a positive regulation (i.e. transcriptional levels positively correlated with gene dosage, clusters 9 and 11), all the enriched biological processes found in cluster 9 were involved in fatty acid metabolism. In cluster 11 significantly enriched terms were related to glycolysis, fatty acid metabolism and anion transport, among others.

### Methanol utilization (Mut) genes are downregulated in strains with increasing ROL copy number, suggesting a Mxr1 titration effect

**Figure 5:**
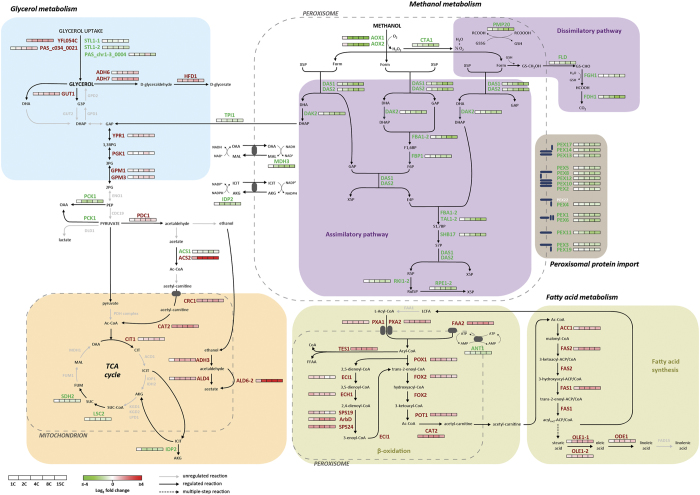
Major metabolic pathways affected by different *ROL* gene dosage in *P. pastoris* cells growing in chemostat cultures using a glycerol/methanol mix as a carbon source. Glycerol uptake pathway (blue), tricarboxylic acid (TCA) cycle (orange), fatty acid metabolism (synthesis and β-oxidation) (brown), methanol assimilation (purple) and peroxisomal protein import (grey) are represented. Gene regulation in each step is indicated by a colour gradient. Unregulated genes are showed in grey.

**Figure 6:**
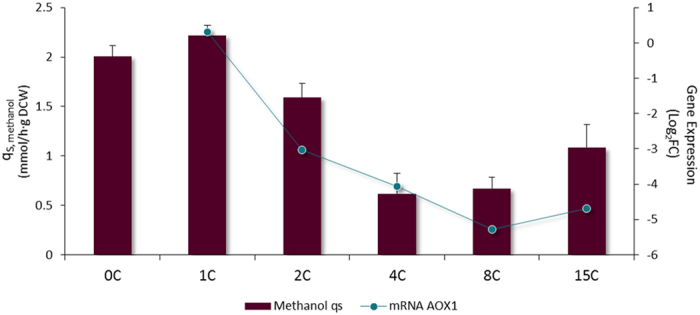
Methanol consumption rate is related to alcohol oxidase expression. In bars, methanol consumption rate of multicopy strains growing in chemostat cultures. Line graph represents the *AOX1* transcript levels measured in the microarrays, comparing to the reference strain 0C. Error bars represent the standard deviation between three independent experiments.

*P. pastoris*, as a methylotrophic yeast, is able to grow on methanol as a sole carbon source. In this study, where cells were grown in continuous cultures using a mixture of glycerol:methanol in the feed medium at conditions ensuring co-assimilation of both carbon sources, a global regulation of the methanol pathway was clearly observed (Fig. 5). Firstly, the oxidation of methanol to formaldehyde, mainly driven by the enzyme alcohol oxidase (encoded by the gene *AOX1*), was downregulated from 8-fold (2C strain) up to 30-fold for the higher *ROL* gene dosage strains compared to the non-producing reference strain. Only in the case of the 1C strain, *AOX1* appeared to be slightly upregulated. *AOX2* was also approximately 5-fold downregulated in all multi-copy strains. Secondly, all the genes involved in the dissimilatory pathway of direct oxidation of methanol to CO_2_ presented a negative transcriptional regulation (glutathione-dependent formaldehyde dehydrogenase *FLD*, S-formylglutathione hydrolase *FGH1*, and formate dehydrogenase *FDH1*). Finally, all the genes of the methanol assimilation pathway, leading to the generation of cell constituents from formaldehyde, were also downregulated (*DAS1, DAS2, TPI1, DAK2, FBA1-2*, and *FBP1*). While the genes encoding for the cytoplasmatic non-oxidative branch of pentose phosphate pathway (*TAL1-1, RKI1-1, RPE1-1*) were not regulated, we observed downregulation of the peroxisomal isoforms of these genes (*TAL1-2, RKI1-2, RPE1-2,* and *SHB17*) in the multi-copy strains along with the genes involved in methanol pathway. Recently, Russmayer and co-workers^3^ reported the presence of a peroxisomal targeting signal (PTS1) in these enzymes, and defined the rearrangement reactions taking place in this peroxisomal xylulose-monophosphate cycle during methanol assimilation in *P. pastoris*. Briefly, xylulose-5-phosphate is regenerated using a set of specialized enzymes localised in the peroxisome, which are only synthetized in the presence of methanol. Furthermore, the overall *ROL*-copy number dependent downregulation of the peroxisomal methanol assimilation pathway might explain the lower biomass yields and reduced methanol consumption capacity observed for the multi-copy strains. In fact, *AOX1* transcriptional levels correlate with the methanol consumption rate profile observed for the series of strains (Fig. 6). As the *ROL* copy number increases in the strains, the methanol assimilation capacity decreases. This results in a significant methanol accumulation in the media (up to 7 g/L, Table 1) of the multi-copy strain cultivations. Coherently, all riboflavin genes involved in the synthesis of the coenzyme flavin adenine dinucleotide (FAD, essential cofactor for alcohol oxidase assembly, and suggested to be regulated by *AOX1* by) were downregulated (*RIB1, RIB3, RIB4, RIB5, FAD1*) in the multi-copy strains. Furthermore, as indicated by Russmayer and co-workers^3^, thiamine pyrophosphate (TPP) is the co-factor of transketolases Das1 and Das2. Consistently, several genes involved in the synthesis of thiamine and TPP were downregulated depending on *ROL* gene dosage (*THI13, THI14, THI20, THI21, THI73* and *THI80*).

**Figure 7:**
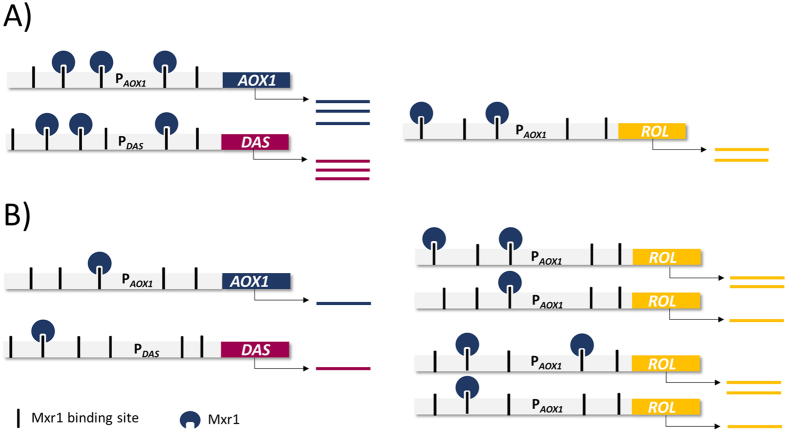
Regulatory model of Mut genes by means of the transcription factor Mxr1 in the presence of methanol. (**A**) Regulation of Mut genes in the 1C strain. (**B**) Regulation of Mut genes in the 4C strain. Due to the numerous Mxr1 binding sites in the promoters of methanol utilization genes such as alcohol oxidase 1 and dihydroxyacetone synthase, and the relative low expression levels of Mxr1, a limitation in the regulation of the Mut genes might be possible in the multicopy strains. Whereas in the 1C strain the number of Mxr1 molecules is enough to allow proper expression of Mut genes, the increase of the number of binding sites in the 4C strain results in lower probability of Mxr1 to bind Mut gene promoters, therefore negatively affecting the methanol assimilation capacity of the strain.

In *P. pastoris* the methanol metabolism is mainly regulated by Mxr1, a transcription factor which is constitutively expressed at low levels, being crucial for the induction of P_*AOX1*_ and P_*DAS*_^6^,^52^,^53^. It seems plausible that the insertion of expression vectors (resulting in an increasing number of P_*AOX1*_ sequences) results in a limitation of free Mxr1 molecules in the multi-copy strains, thereby negatively affecting the transcriptional levels of methanol-induced genes, including *ROL*. As it is proposed in Fig. 7A, the low expression of Mxr1 would be enough to accomplish for the activation of P_*AOX1*_ and P_*DAS*_ in the single copy strain and consequently to properly metabolize methanol as a carbon source. However, in the case of the 4C strain (Fig. 7B), the distribution of Mxr1 among the multiple binding sites would be insufficient to achieve a regular expression of *DAS* and *AOX1*, resulting in a reduction of methanol assimilation. However, this hypothesis would not explain the fact that even though *ROL* mRNA levels are constant for the high copy strains, a decrease in extracellular lipase activity is observed (Fig. 1A). In fact, low amounts of intracellular lipase point to a translational arrest rather than a strong UPR response as the reason of the observed decrease of Rol production in the strains 4C, 8C and 15C, as recently reported by Edwards-Jones and co-workers^54^ for multi-copy strains expressing human trypsinogen. Briefly, these authors observed in *P. pastoris* fed-batch cultures that in the multi-copy strain transcripts were not being translated and, consequently, a decrease in traffic though ER was detected compared to the single copy strain. This correlates with our observations that not only extracellular, but also intracellular Rol levels (in the insoluble fraction) were lower in the 8C and 15C strains. Also, this phenomenon was accompanied by a slow adaptation to methanol metabolism, consistent with our hypothesis of a Mxr1 limitation in the multi-copy strains. As in our case, no evidences of UPR were found in the multi-copy strains, suggesting the high metabolic demand for recombinant protein synthesis as the major cause of translational arrest.

Several studies in yeast (*S. cerevisiae*) and filamentous fungi (*Aspergillus niger*) demonstrate that the increase of the heterologous gene expression cassettes beyond a certain limit it is not advantageous in terms of protein yields because essential transcription factors become limiting^55^. Since *MXR1* is constitutively expressed at low levels in *P. pastoris*, it is plausible that Mxr1 is available in limiting amounts and we can therefore expect a “titration effect” of free molecules of this transcription factor as the number of Mxr1-DNA binding regions increase. Indeed, preliminary data from our group^56^ reveals that overexpression of an engineered form of Mxr1^57^ can improve transcriptional levels of *ROL* and other methanol related genes in the 4C strains, resulting in a substantial improvement of methanol consumption and extracellular Rol levels.

### Biogenesis of peroxisomes is downregulated in strains with higher ROL gene dosage

Peroxisomes are crucial organelles in *P. pastoris* that accomplish three main functions: (i) host enzymes needed for methanol metabolization through the assimilation pathway, (ii) provide a compartment to confine and eliminate hydrogen peroxide produced by alcohol oxidase, and (iii) harbour the fatty acid β-oxidation pathway^58^,^59^. In this study, methanol metabolism was entirely downregulated in multi-copy strains. Accordingly, we observed a global regulation of peroxin genes, encoding for proteins involved in peroxisome biogenesis. On one hand, *PEX1, PEX3, PEX6, PEX11C, PEX19, PEX25* and *PEX30*, involved in the biogenesis of peroxisome membrane proteins (from ER or by fission^60^) were downregulated, especially in the higher multi-copy strains, 8C and 15C. On the other hand, *PEX2, PEX4, PEX5, PEX8, PEX10, PEX12, PEX13, PEX14* and *PEX17*, which are genes functionally related with protein import to the peroxisomal matrix, were also downregulated (Fig. 5). Peroxisomal matrix proteins contain specific peroxisomal targeting signals (PTS1 or PTS2) that are post-translationally recognized in the cytosol by the import receptors Pex5 and Pex7, respectively^61^. Notably, *PEX5*, was downregulated, in agreement with the downregulation of the key genes of the methanol pathway (*AOX1, CTA1, DAS1* and DAS2), all encoding PTS1 proteins^62^. Conversely, no regulation was detected in the gene *PEX7*, coherent with previous observations^3^,^63^, supporting the hypothesis that methanol metabolism is not dependent on the PTS2 import pathway. Strikingly, *ATG8* and *ATG18*, involved in pexophagy (i.e. autophagy of peroxisomes)^64^ presented a negative regulation compared to the non-producing strain. According to Vanz and co-workers^4^, a constitutive recycling of peroxisomes through pexophagy is present in methanol cultures to deal with peroxisomes damaged by reactive oxygen species (ROS). Consistently, ROS levels detected in these series of *ROL* strains growing under steady-state chemostats in analogous conditions were very low^16^. Similarly, Hesketh and co-workers^28^ described the acclimation of producing strains growing in sorbitol-methanol chemostat cultures, where only a transient oxidative stress response was detected upon the onset of inducing conditions (e.g. switch from non-inducing (sorbitol only) to inducing (sorbitol + methanol) feed in continuous cultures), but not under steady state growth conditions. Overall, low ROS levels, the lack of regulation of oxidative stress marker genes and the downregulation of the *CTA1* gene encoding for the enzyme catalase, responsible for the degradation of hydrogen peroxide in the peroxisomes, suggests that oxidative stress has a minor role in the physiological adaptation to higher *ROL* gene dosage during steady-state growth in chemostats. However, we cannot exclude that ROS levels increase transiently upon methanol induction, as suggested by previous analyses of this series of strains in shake flask cultures^16^. Furthermore, *HEM1*, the gene encoding the first step in heme synthesis (the cofactor of catalase), and *PET18*, coding for a heme oxygenase-like protein (that catalyses the degradation of heme) were downregulated in a *ROL* copy dependent manner. According to Russmayer and co-workers^3^, iron metabolism is closely related to catalase detoxification and methanol metabolism. In our case, several genes coding for iron permeases and ferric permeases, among other genes related to iron metabolism, were upregulated in the producing strains (*FET3, FET4*-2, *FRE2, FRE3, FRE6, FTR1, SIT1-2*) in contrast to the overall downregulation of the Mut genes.

### Fatty acid metabolism is strongly altered, showing upregulation of both biosynthetic and β-oxidation pathways in Rol producing strains

In yeast, β-oxidation (the main pathway for fatty acid degradation) is performed exclusively in the peroxisomes. The generated acetyl-CoA can be redirected to the glyoxylate cycle or transported to the mitochondria via the carnitine shuttle, being a source of energy and anaplerotic supply of building blocks. Briefly, medium chain fatty acids (MCFA) are transported into peroxisomes by diffusion and are activated by the acyl-CoA synthetase Faa2. In the case of long chain fatty acids (LCFA), the heterodimer formed by Pxa1-Pxa2 is required to enter into the peroxisomes. As suggested by Van Roermund and co-workers^65^, Pxa1-Pxa2 also accepts activated LCFAs, which are subsequently re-esterified by acyl-CoA synthetase Faa2 or Fat1. The β-oxidation process begins with the oxidation of acyl-CoA substrates by Pox1. Further reactions are catalysed by multifunctional enzyme Fox2, generating hydroxyacyl-CoA and 3-ketoacyl-CoA. The final reaction, driven by the 3-ketoacyl-CoA thiolase Pot1, results in a molecule of acetyl-CoA and a C2 shortened acyl-CoA, that can re-enter in the β-oxidation cycle again. A global upregulation of all the β-oxidation steps (*FAA2, PXA1, PXA2, FAT1, POX1, FOX2* and *POT1* genes) was observed in all producing strains, as depicted in Fig. 5. Even *TES1*, encoding for the acyl-CoA thioesterase involved in the maintenance of the pool of free CoA inside the peroxisome^66^, was significantly upregulated in all the strains. Unlike the methanol pathway, this global regulation of all the catabolic fatty acid related genes does not seem to be *ROL*-copy dependent, that is, it is similarly affected in all the producing strains. Genes encoding β-oxidation of unsaturated fatty acids with trans and cis double bonds at odd-numbered positions or cis double bonds at even positions (i.e. oleic acid) were also overall upregulated, but with lower magnitude.

The acetyl-CoA generated from β-oxidation has two ways to be exported from peroxisomes: Firstly, through the conjugation to carnitine mediated by the carnitine acetyltransferase Cat2 and subsequent transport to mitochondria, where it binds to the carrier protein Crc1. The second possible destination of acetyl-CoA is the glyoxylate cycle, generating succinate as a final product^67^. The weak regulation of the genes encoding for enzymes involved in glyoxylate cycle, along with significant upregulation of *CAT2* and *CRC1* in all the producing strains might indicate that acetyl-CoA is redirected mainly to the mitochondria, at the expense of the glyoxylate cycle. Because peroxisomes are organelles impermeable to dinucleotides (i.e. FAD, NAD^+^, NADPH) and acetyl-CoA^68^, they depend on several shuttles to maintain the cofactor pool. In the case of FAD-containing enzymes (i.e. acyl-CoA oxidase, alcohol oxidase), they are assembled with the cofactor in the cytosol, and subsequently reoxidised into the peroxisome, generating H_2_O_2_^69^. Conversely, intraperoxisomal NAD^+^ is regenerated through a malate/oxaloacetate shuttle, via peroxisomal malate dehydrogenase Mdh3, while reduction of NADP^+^ might be performed by the 2-ketoglutarate/isocitrate shuttle, as suggested for *S. cerevisiae*^67^. In both cases, all the corresponding genes were negatively regulated when compared to the non-expressing control strain (Fig. 5). Similarly, the adenine nucleotide transporter encoded by *ANT1* (coupled to the activation of MCFAs by *FAA2* gene) was also downregulated, along with the catalase *CTA1*.

Remarkably, also fatty acid biosynthesis was significantly upregulated when *ROL* gene dosage increased. Both acetyl-CoA carboxylase *ACC1* gene and the fatty acid synthase complex (encoded by *FAS1* and *FAS2*) were upregulated, particularly in the strains harbouring more than 1 *ROL* copy. Furthermore, three out of the four fatty acid desaturase genes described in *P. pastoris*^70^ were upregulated (*OLE1-1, OLE1-2, ODE1*). Total fatty acid composition of the strains was further analysed to study the effect of desaturase regulation on the fatty acid profile (Supplementary Table S9). The results confirmed that the most abundant fatty acids in *P. pastoris* were oleic acid, linoleic acid, palmitic acid, linolenic acid and stearic acid, as it has been previously reported^70^,^71^. Interestingly, it seems that in producing strains the most abundant fatty acid was linoleic acid, unlike the 0C reference strain, in which oleic acid was the main fatty acid. The upregulation of the gene *ODE1* coding for desaturase Fad12, which catalyses the synthesis of linoleic acid with oleic acid as substrate, could explain this change in the fatty acid profile. In addition, the total fatty acid content of each strain was correlated negatively with their corresponding number of *ROL* copies, especially in the multi-copy strains with higher *ROL* gene dosages.

Increased transcriptional levels of genes encoding both fatty acid synthesis and β-oxidation could reflect an increased turnover of fatty acids, as previously reported in *S. cerevisiae*^72^. This evidence is reinforced by the fact that the gene encoding for the major contributor to the synthesis of triacylglycerols, the acyltransferase Dga1, is downregulated, and no other genes involved in lipid metabolism (i.e. sphingolipids, phospholipids) are regulated. Therefore, it seems that a futile cycle involving acetyl-CoA is promoted in the producing strains. As it was mentioned before, a high upregulation of the gene *ACS2* was observed in the multi-copy strains. This acetyl-CoA synthetase is the responsible for the synthesis of nucleocytosolic acetyl-CoA in budding yeast, and as it was reported by Eisenberg and co-workers^73^, an hyperactivation of this enzyme can lead to histone acetylation and subsequent repression of autophagy genes, consistent with the downregulation of the pexophagy genes *ATG8* and *ATG18* mentioned above. On the other hand, regulation of acetyl-CoA is also performed by *de novo* synthesis of fatty acids^74^, competing with protein acetylation for the same pool of acetyl-CoA. Altogether, these results point to a complex mechanism of regulation at transcriptional and translational level. Alternatively, the strong regulation of fatty acid metabolism might reflect a limited availability of acetyl-CoA as a result of its increased anabolic demand as a building block (e.g. for membrane lipid synthesis needed to increase secretory capacity), as well as for fuelling the TCA cycle, i.e. for energy production, as recently suggested by Klein and co-workers^75^. However, as it was mentioned above, no regulation of the membrane lipid biosynthetic pathways was detected in the Rol producing strains.

### Impact of ROL gene dosage on amino acid metabolism, glycerol and energy metabolism, protein synthesis, folding, secretion and degradation pathways is rather limited or not significant

Although an impact on amino acid metabolism due to recombinant protein requirements was expected^76^, no overall regulation was detected in the different amino acid biosynthetic pathways and for amino acid permeases, except for the lysine, with a slight downregulation of some of the steps in the multi-copy strains (*LYS1, LYS2, LYS4, LYS20, MKS1*). Interestingly, *LYP1* and PAS_chr2-1_0649, coding for lysine permeases, were upregulated compared to the reference strain. Consistently, no ribosome associated genes were significantly regulated (Supplementary File S2). Even though higher glycerol consumption rates were observed as the *ROL* copy number increased, only two putative glycerol permeases (genes *YFL054C* and PAS_c034_0021) were upregulated in the multi-copy strains. Notably, although previous studies had revealed increased metabolic activity (flux) of the TCA cycle to meet higher energy requirements (NADH, ATP) due to recombinant protein production^23^,^76^, only *CAT2* (essential for carnitine acetyltransferase-mediated transport of cytosolic acetyl-CoA to the mitochondria) and the citrate synthase encoding gene (*CIT1*) appeared to be slightly upregulated in multi-copy strains.

**Figure 8:**
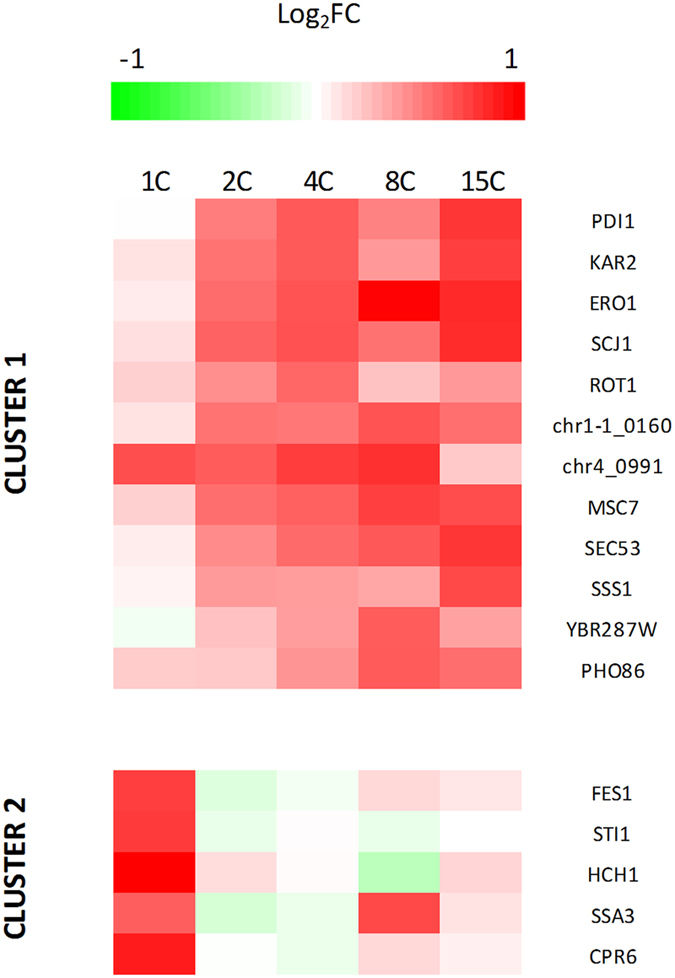
Regulation of genes involved in folding and secretion. A cluster analysis was performed using a *k-means* algorithm (k = 2). Cluster 1 is mainly comprised of ER resident proteins, whereas cluster 2 contains cytosolic chaperones.

As already mentioned above, approximately 30% of total Rol was found in the insoluble cell fraction of all the producing strains (Supplementary Table S4). To study the relationship between this lipase retention and the UPR, secretion and folding genes were analysed. As shown in Fig. 8, a total of 17 genes were regulated in at least one strain compared to the non-producing control. Regulation of these genes was not intense, with a maximum of a 2-fold change. Cluster analysis was performed, and two main groups were obtained. One cluster exhibited an upregulation in the multi-copy strains, whereas there was no regulation in the single copy strain (except for PAS_chr4_0991). Functional analysis revealed that this set of genes were ER resident proteins, mainly chaperones and protein disulphide isomerases. The second cluster comprised genes only regulated in the single copy strain (except *SSA3*). In this case, all the genes encoded cytosolic heat shock proteins involved in folding. Although no regulation of the transcription factor *HAC1* (main regulator of UPR) was observed, other genes commonly used as UPR markers (e.g. *KAR2, PDI*) presented a relative low upregulation (2-fold comparing with the non-producing strain) confirming that this stress response does not seem to be the major bottleneck in the Rol-producing strains growing under steady-state conditions. Additionally, no global regulation was found for vacuolar proteases or the ERAD pathway genes (Supplementary File S2), altogether indicating that ER stress was not globally activated in the multi-copy strains.

## Conclusions

Increasing the heterologous gene dosage is a well-established strategy to obtain *P. pastoris* strains producing higher amounts of recombinant proteins. However, this strategy often reveals clear limitations when the recombinant gene dosage is increased beyond a certain threshold, causing an important physiological impact to the producing cells.

This transcriptomics study helps to understand the physiological adaptations of cellular mechanisms to increased heterologous gene dosages when the strong, methanol-inducible *AOX1* promoter is used. Remarkably, the expression system appeared to be strongly limited at the transcriptional level, rather than at the energy and building block supply chain or the protein folding and secretion pathways. Specifically, the transcriptional attenuation of methanol utilization genes in multi copy strains, including P_*AOX1*_ used to drive heterologous gene expression, resulted in a strong downregulation of methanol metabolism, peroxisome biogenesis and fatty acid metabolism. This further impacted growth parameters and productivities of the multi-copy strains growing in glycerol:methanol mixtures (higher glycerol consumption and reduced methanol consumption rates). In addition, our data also support evidence for a decrease in translation that may also be contributing to the lower protein secretion levels observed at higher gene dosages.

This work provides a comprehensive data set for the understanding of limitations of P_*AOX1*_-based systems when developing strains with an increased number of heterologous gene copies, and will have a major impact in the design of novel cell engineering strategies to generate high-producing strains. Nonetheless, further studies on cellular responses that may be specific to higher gene dosages (e.g. ≥15 *ROL* copies) may provide additional basis for the identification of novel targets for strain engineering.

## Additional Information

**How to cite this article:** Cámara, E. *et al*. Increased dosage of *AOX1* promoter-regulated expression cassettes leads to transcription attenuation of the methanol metabolism in *Pichia pastoris. Sci. Rep.* **7**, 44302; doi: 10.1038/srep44302 (2017).

**Publisher's note:** Springer Nature remains neutral with regard to jurisdictional claims in published maps and institutional affiliations.

## Supplementary Material

Supplementary Information

Supplementary File S2

Supplementary File S5

Supplementary File S6

Supplementary File S7

Supplementary File S8

## Figures and Tables

**Table 1:** Reconciled macroscopic growth parameters of Rol-producing and reference strain in glycerol:methanol chemostat cultures.

	q_s, glycerol_ (mmol g^−1^ DCW h^−1^)	q_s, methanol_ (mmol g^−1^ DCW h^−1^)	q_CO2_ (mmol g^−1^ DCW h^−1^)	q_O2_ (mmol g^−1^ DCW h^−1^)	DCW (g^−1^ L^−1^)	Yx/s (g^−1^ DCW C-mol^−1^)	Residual methanol (g/L)	Residual glycerol (g/L)
**0C**	−1.12 ± 0.01	−2.01 ± 0.11	2.03 ± 0.10	3.52 ± 0.14	12.82 ± 0.16	16.84 ± 0.53	0.11 ± 0.10	0.04 ± 0.02
**1C**	−1.20 ± 0.03	−2.22 ± 0.10	2.35 ± 0.13	3.98 ± 0.06	12.03 ± 0.11	15.97 ± 0.18	0.10 ± 0.09	0.03 ± 0.01
**2C**	−1.51 ± 0.03	−1.59 ± 0.14	2.28 ± 0.09	4.16 ± 0.24	8.99 ± 0.15	14.35 ± 0.08	4.39 ± 0.28	0.04 ± 0.01
**4C**	−1.71 ± 0.09	−0.62 ± 0.21	2.08 ± 0.28	3.82 ± 0.31	8.66 ± 0.41	16.18 ± 1.04	7.07 ± 0.19	0.03 ± 0.03
**8C**	−1.75 ± 0.05	−0.67 ± 0.12	1.97 ± 0.08	3.75 ± 0.06	8.26 ± 0.10	15.37 ± 0.14	7.52 ± 0.23	0.03 ± 0.1
**15C**	−1.73 ± 0.12	−1.08 ± 0.24	2.18 ± 0.16	4.05 ± 0.23	8.55 ± 0.34	14.92 ± 0.13	5.90 ± 0.92	0.02 ± 0.02

Substrate consumption and biomass production rates for each strain. Consistency index h was below 5 in all cases (95% confidence level). Data are shown as means ± standard deviation based on three independent experiments for each strain. q_s_: reconciled substrate consumption specific rate; qCO_2_: CO_2_ exchange rate; qO_2_: O_2_ oxygen uptake rate; Y_X/S_: reconciled biomass/substrate yield.
