# Supplementary material for: Increased dosage of AOX1 promoter-regulated expression cassettes leads to transcription attenuation of the methanol metabolism in Pichia pastoris
Source: Sci Rep. 2017 Mar 15;7:44302. doi: 10.1038/srep44302 (PMC5353721; doi:10.1038/srep44302)
Supplement: Supplementary Information [file srep44302-s1.pdf]

Increased dosage of *AOX1* promoter-regulated expression cassettes leads to transcription attenuation of the methanol metabolism in *Pichia pastoris*

Elena Cámara[1], Nils Landes[2,3], Joan Albiol[1], Brigitte Gasser[2,3], Diethard Mattanovich[2,3], Pau Ferrer[1*]

[1]Department of Chemical, Biological, and Environmental Engineering, Escola d'Enginyeria, Universitat Autònoma de Barcelona, Bellaterra (Cerdanyola del Vallès) 08193, Catalonia, Spain;

[2]Department of Biotechnology, BOKU - University of Natural Resources and Life Sciences Vienna, Muthgasse 18, 1190 Vienna, Austria

[3]Austrian Centre of Industrial Biotechnology, A-1190 Vienna, Austria

*Corresponding author: pau.ferrer@uab.cat

Supplementary Table S1. Primer sequences used for ddPCR. Tm, melting temperature; %GC, guanosine + cytosine content (in %) of the oligonucleotides.

OLIGONUCLEOTIDE	SEQUENCE	Tm	% GC	AMPLICON SIZE (bp)
ROL fwd primer	5’ – AAGTGGGACTGTGTCCAATG – 3’	61	50	158
ROL rev primer	5’ – GCACTTCTGAAGGAGTTG – 3’	57	50	
ACT1 fwd primer	5’ – CACCACACCTTCTACAAC – 3’	56	50	139
ACT1 rev primer	5’ – AGAAGGCTGGAACGTTG – 3’	58	52	

Supplementary Table S3. Additional physiological parameters

	Lipase activity (UA/mL)	OD_{600}	Biomass (mmol g-1 DCW h-1)	Yx/s (g biomass g^{-1} substrate	Respiratory Quotient (RQ)
**0C**	n.d.	55.44 ± 2.14	3.32 ± 0.13	0.56 ± 0.01	0.57 ± 0.05
**1C**	626.9 ± 139.0	52.44 ± 1.71	3.44 ± 0.17	0.58 ± 0.02	0.59 ± 0.03
**2C**	3922.3 ± 376.1	41.22 ± 1.58	3.65 ± 0.04	0.61 ± 0.01	0.55 ± 0.01
**4C**	3368.3 ± 329.2	37.11 ± 0.69	3.50 ± 0.18	0.65 ± 0.03	0.54 ± 0.03
**8C**	1831.5 ± 153.6	36.67 ± 0.33	3.75 ± 0.12	0.64 ± 0.00	0.53 ± 0.03
**15C**	1455.1 ± 66.8	36.78 ± 0.51	3.87 ± 0.11	0.63 ± 0.03	0.54 ± 0.03

Supplementary Table S4. Rol content quantification. Data are shown as means ± standard deviation based on triplicate measurements (one for each chemostat performed). *$P < 0.05$ (compared to reference strain in the case of protein content, compared to the single copy strain in the rest of cases). SN, supernatant; IF, insoluble fraction; SF, soluble fraction.

	**SN protein content (mg protein/g DCW)**	**SN Rol content (µg Rol/g DCW)**	**IF Rol content (µg Rol/g DCW)**	**SN Lipase specific activity (UA/µg Rol)**	**SF Lipase specific activity (UA/mg protein)**
0C	1.74 ± 0.41	-	-	-	-
1C	1.70 ± 0.03	24.16 ± 13.13	14.93 ± 3.65	29.08 ± 10.18	0.09 ± 0.03
2C	2.56 ± 0.19 *	175.20 ± 18.90 *	76.01 ± 10.83 *	22.64 ± 3.91	0.29 ± 0.05 *
4C	2.69 ± 0.08 *	146.86 ± 29.38 *	63.33 ± 17.05 *	23.62 ± 5.90	0.30 ± 0.07 *
8C	2.24 ± 0.11 *	52.54 ± 7.66 *	35.29 ± 9.69 *	28.93 ± 2.97	0.23 ± 0.05 *
15C	2.29 ± 0.08 *	97.00 ± 3.03 *	58.55 ± 13.39 *	18.35 ± 2.64	0.27 ± 0.03 *

Supplementary Table S9. Total fatty acid composition of Rol-producing and reference strains. Fatty acid content was quantified by gas chromatography coupled with mass spectrometry. Only the 5 main fatty acids detected are represented. Data are shown as means ± standard deviation based on three independent experiments for each strain. * indicates $P < 0.05$ compared to 0C.

	% oleic acid (C18:1)	% linoleic (C18:2n-6)	% palmitic (C16:0)	% α-linolenic (C18:3n-3)	%stearic (C18:0)	TOTAL (µg FA/mg DCW)
0C	35.3 ± 3.7	26.9 ± 2.6	13.6 ± 1.3	10.8 ± 0.1	2.1 ± 0.1	45.9 ± 4.2
1C	28.2 ± 1.9 *	31.6 ± 2.1 *	11.9 ± 0.9 *	12.9 ± 0.1 *	1.6 ± 0.4	40.1 ± 2.9
2C	28.4 ± 0.7 *	31.4 ± 1.8 *	11.7 ± 0.5 *	10.4 ± 0.1	2.5 ± 0.5	40.2 ± 1.4
4C	28.2 ± 3.1 *	31.6 ± 2.1 *	12.0 ± 1.2 *	11.1 ± 0.1	2.1 ± 0.3	35.6 ± 2.7 *
8C	26.2 ± 1.5 *	32.0 ± 2.3 *	13.6 ± 1.1	13.5 ± 0.2 *	2.8 ± 0.3 *	35.9 ± 2.5 *
15C	26.5 ± 0.7 *	30.8 ± 0.2 *	14.0 ± 0.7	12.0 ± 0.5	2.7 ± 0.2 *	32.5 ± 5.2 *
