# Supplementary material for: Increased dosage of AOX1 promoter-regulated expression cassettes leads to transcription attenuation of the methanol metabolism in Pichia pastoris
Source: Sci Rep. 2017 Mar 15;7:44302. doi: 10.1038/srep44302 (PMC5353721; doi:10.1038/srep44302)

Supplementary File S5. Additional transcriptomic analysis of regulated genes in *ROL* strains.

A) Venn diagrams showing the number of specific or common genes for among 1C strain (producing control), 2C strain (best producer) and 15C strain (highest copy number. B) and C) Principal component analysis (PCA) of the expression profile data. Regulated genes are represented as dots (observations), whereas red lines indicated the projection trend of each strain. The first (PCA1) and the second (PCA2) principal components are represented in B), whereas the second and the third (PCA3) are represented in C).

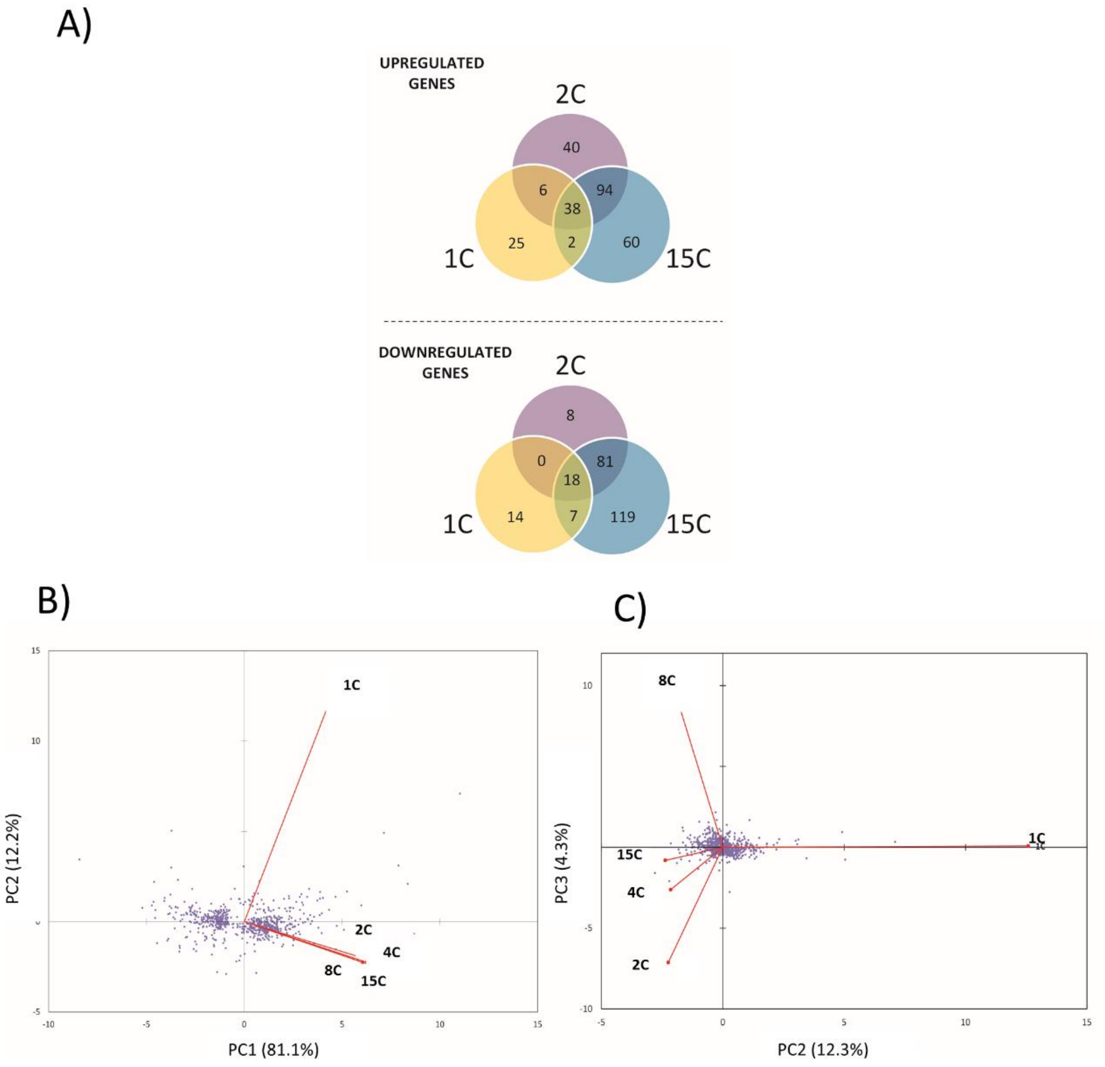

Supplement: Supplementary File S5 [file srep44302-s3.pdf]
